# Comparative outcomes of tuina therapy combined with functional exercise versus conventional physical therapy in infants with congenital muscular torticollis: a retrospective exploratory study

**DOI:** 10.3389/fmed.2026.1869096

**Published:** 2026-06-29

**Authors:** Xianzhu Tian, Chaowen Zhu, Zhen Zhu, Zixuan Nie

**Affiliations:** 1 Jiujiang Traditional Chinese Medicine Hospital, Jiujiang, China; 2 Jiujiang Maternity and Child Health Care Hospital, Jiujiang, China

**Keywords:** cervical range of motion, congenital muscular torticollis, functional exercise, infant rehabilitation, physical therapy, retrospective study, sternocleidomastoid, Tuina therapy

## Abstract

**Background:**

Congenital muscular torticollis (CMT) is a common pediatric musculoskeletal condition managed with conservative physical therapy. Traditional Chinese Medicine Tuina therapy is used adjunctively in some settings, but high-quality comparative evidence is limited.

**Objective:**

To compare short-term clinical outcomes in infants with CMT who received combined Tuina therapy and functional exercise (integrated group) versus conventional physical therapy (control group) during routine care.

**Methods:**

Anonymized clinical records of 120 infants treated between January and June 2024 were retrospectively reviewed. Sixty infants received integrated Tuina–functional exercise and 60 received conventional physical therapy. Treatment allocation was non-randomized. Primary outcomes were cervical range-of-motion (ROM) deficit, sternocleidomastoid (SCM) muscle thickness ratio, and head tilt angle at 12 weeks. Between-group differences were analyzed using independent-samples *t*-tests, and 95% confidence intervals (CIs) and Cohen’s d were reported. This study is reported in accordance with the STROBE statement.

**Results:**

Baseline characteristics were comparable between groups. At 12 weeks, the integrated group demonstrated greater mean change from baseline in ROM deficit (−25.9 ± 7.9° vs. −21.4 ± 9.3°; difference 4.4°, 95% CI 1.3–7.5, *p* = 0.006, *d* = 0.51), SCM thickness ratio (−1.35 ± 0.49 vs. −1.04 ± 0.55; difference 0.31, 95% CI 0.13–0.50, *p* = 0.001, *d* = 0.60), and head tilt angle (−19.8 ± 5.1° vs. −16.1 ± 5.2°; difference 3.7°, 95% CI 1.9–5.6, *p* < 0.001, *d* = 0.72). Home-program adherence was significantly higher in the integrated group (85.7 ± 11.2% vs. 78.0 ± 15.2%; *p* = 0.002). No serious adverse events were documented.

**Conclusion:**

The combined Tuina–functional exercise program was associated with significantly greater improvements in all primary outcomes compared with conventional physical therapy. However, given the retrospective, non-randomized design and unequal therapist-contact time, findings should be considered exploratory. Prospective randomized controlled trials with blinded assessment and standardized treatment intensity are required.

## 1 Introduction

Congenital muscular torticollis (CMT) is a relatively frequent musculoskeletal disorder in children that is characterized by the ipsilateral shortening or fibrosis of the sterna cleavage (SCM) muscle, which leads to ipsilateral cervical lateral flexion, contralateral cervical rotation, and limited movement of the neck (1, 2). It has an incidence of 0.3–3.9% of live births and perinatal factors such as birth trauma, primiparity, and intrauterine constraint have been identified as risk factors (3). Unidentified and untreated, CMT can be linked to craniofacial asymmetry, positional plagiocephaly, motor asymmetry, and musculoskeletal comorbidities, such as developmental dysplasia of the hip (1, 4). These possible sequelae highlight the clinical significance of early and effective intervention especially at the stage of high neuromuscular plasticity in early infancy.

The traditional management of CMT is mostly conservative physical therapy that includes passive SCM stretching, active cervical range-of-motion (ROM) exercises, positioning, environmental adaptation, caregiver education, and structured exercise programs at home (1, 5, 6). Even though the early-initiation physical therapy is closely linked with positive outcomes, the response to treatment is still uneven. Some percentage of babies will need extended intervention beyond several months, and a smaller group will need surgery release (7). Such restrictions underscore the clinical importance of investigating adjunctive or integrative treatment approaches that can potentially augment outcomes and shorten the treatment scope.

Traditional Chinese Medicine (TCM) Tuina therapy is a unified manual therapy that involves systematic soft-tissue manipulation, acupoint stimulation and rhythmic mobilization. It has been considered as a complementary therapy to CMT, and systematic reviews show that it may have an impact on cervical ROM and SCM symmetry, but the evidence is weak because of the variations in protocols, sample size and outcome measures (8–10). Tuina is a safe method and its addition to multimodal rehabilitation programs has been proposed as a clinically feasible add-on method (11).

Modern rehabilitation strategies increasingly prioritize active, functional approaches. Functional exercise interventions for CMT include visual fixation, symmetrical postures, prone play, head righting, and parent-facilitated active movement to enhance postural alignment and motor skill development (1, 12). Recent research indicates that passive manual therapy combined with active exercise may be more effective than using either approach alone (6).

While there is increasing clinical interest in combined therapies, there is a paucity of research evaluating systematic combinations of Tuina therapy and standardized functional exercise in clinical practice using objective longitudinal outcome measures. Therefore, the current retrospective exploratory study will examine and compare observed clinical effects of a combination of Tuina therapy and functional exercise with standard physical therapy in infants with CMT over 12 weeks. The observational nature of this non-randomized study is aimed to be exploratory and generate hypotheses for future prospective controlled studies.

## 2 Literature review

### 2.1 Pathophysiology of congenital muscular torticollis

Congenital muscular torticollis (CMT) involves unilateral fibrosis, hypertrophy or contracture of the SCM muscle, causing abnormal head positioning and neck restriction (1, 13). Its causes are complex and include intrauterine mechanical restriction, birth trauma, postural preference and other musculoskeletal conditions (3, 4). SCM thickness and asymmetry are objectively evaluated with the help of ultrasound, and the ratios of SCM thickness have been demonstrated as objective clinical parameters in the diagnosis and treatment of CMT (14).

There are also neuromotor components in CMT such as head control asymmetry, postural responses and symmetrical movement retardation. Predictors of treatment are the time of diagnosis, the clinical severity at presentation, the presence or absence of a mass or pseudotumor in the SCM and the time of intervention. The faster recovery and the ability to resolve the condition is always associated with the early diagnosis, in particular, in the first 3 months of life, which can be explained by the flexibility of the soft tissues of the newborn and his/her neuroplasticity (1, 5).

### 2.2 Conventional physical therapy

The primary non-surgical treatment of CMT is traditional physical therapy, which is supported by clinical practice guidelines (1). Conventional interventions are passive SCM stretching, active cervical ROM, positioning, environmental adaptations, caregiver education, and home exercise (6). Age at treatment, initial severity, compliance and adherence to home programs by caregivers are factors that influence clinical outcomes (7). Song et al. (14) demonstrated objective evidence of the effectiveness of conventional physical therapy in a randomized clinical trial by demonstrating better SCM thickness ratio and head rotation under the influence of physical therapy intervention.

### 2.3 Tuina therapy

Tuina therapy within TCM involves structured soft-tissue manipulation, acupoint stimulation, and rhythmic mobilization intended to improve soft-tissue flexibility, local circulation, and neuromuscular regulation. A systematic review and meta-analysis by Chen et al. (8) reported that TCM massage improved cervical ROM and resolution rates in CMT, though overall evidence quality was limited by methodological weaknesses and heterogeneous protocols. Kim et al. (9) further demonstrated additive effects of combining Tuina with herbal medicine in a meta-analysis. Tang et al. (10) reported in a retrospective comparative study that massage therapy was associated with improved cervical ROM and functional outcomes with no major adverse events. Lin et al. (11) additionally demonstrated favorable outcomes from massage combined with medium-frequency electrotherapy in infants with CMT. Despite a favorable safety profile, high-quality randomized controlled trials with standardized protocols remain needed (7).

### 2.4 Functional exercise approaches

Functional exercise approaches emphasize active, movement-based interventions targeting neuromotor control, postural symmetry, and age-appropriate motor development. Interventions include head control, eye tracking, prone positioning, symmetrical handling, parent-supported active play (1, 12). The Muscle Function Scale (MFS) (15), an ordinal 0–4 scale to assess lateral neck flexor function in infants, supports the reporting of functional outcomes in studies of CMT. While passive stretching has been shown to improve motor asymmetry and limited function, it may not be sufficient to improve function, suggesting multimodal approaches should be considered (6).

## 3 Methodology

### 3.1 Study design

This was a retrospective exploratory study using anonymized secondary data from infants with congenital muscular torticollis (CMT). No recruitment of patients, randomization, experimental treatment, or contact with participants was undertaken. The study was retrospective and observational, and the results are descriptive and exploratory. The manuscript was written in accordance with STROBE guidelines, as appropriate. As the study involved only retrospective review of fully anonymized clinical records with no patient contact or intervention, formal ethical approval was not required under the applicable institutional policy; however, institutional awareness and administrative approval for use of the anonymized dataset was obtained prior to data access. All data were handled in accordance with institutional data governance and privacy standards.

### 3.2 Participants

Inclusion criteria required a clinical diagnosis of primary CMT based on SCM shortening, restricted cervical mobility, and postural asymmetry, consistent with current guideline-based descriptions (1). Infants were included if aged 0–12 months at presentation and if complete baseline and 12-week primary outcome data were available. Infants were excluded if they had secondary torticollis attributable to neurological, ocular, skeletal, or syndromic causes; had received prior CMT treatment;.had a ROM deficit < 10° and therefore did not meet the clinical threshold for intervention; or had incomplete primary outcome data in their clinical record.

For exploratory subgroup analysis, baseline clinical severity was operationally classified as: Mild (ROM deficit < 15° and SCM thickness ratio < 1.5); Moderate (ROM deficit 15°–30° or SCM thickness ratio 1.5–2.5); or Severe (ROM deficit > 30° or SCM thickness ratio > 2.5). These categories were study-specific, were not externally validated, and should be interpreted as exploratory.

### 3.3 Sample size

The sample size was not determined by a power calculation but rather the number of eligible cases available during the study period (retrospective exploratory study). All infants who were eligible and treated in the study period were enrolled. A total of 120 infants (*n* = 60 per group) were included. *Post hoc* sensitivity calculations indicated that this sample provided approximately 80% power to detect a between-group difference of 6° in ROM deficit assuming a standard deviation of approximately 12° at α = 0.05 (two-sided). Because the sample was not prospectively planned, all findings should be interpreted cautiously.

### 3.4 Treatment allocation

The treatment was not randomly allocated. Infants were assigned to either one of two approaches to standard clinical care depending upon departmental policy, the choice of the clinician, parental preference and service availability. The non-random allocation technique does not exclude the risk of selection bias and confounding. Although the baseline characteristics were matched, other unmeasured variables like motivation of the caregivers, socioeconomic status, and health literacy might have been different, thus influencing the adherence and outcomes.

### 3.5 Interventions

#### 3.5.1 Integrated group: Tuina therapy combined with functional exercise

The infants in the integrated group received 12 weeks of combined therapy, three sessions per week, with each session lasting 45 min. Sessions were structured as follows: Tuina therapy (approximately 20 min), functional exercise (approximately 15 min), and caregiver education and home-program training (approximately 10 min). Progression criteria were applied weekly: session intensity and exercise difficulty were advanced when the infant tolerated the current level without distress and demonstrated measurable improvement in active ROM during in-session observation.

Tuina treatment was provided by qualified TCM practitioners with pediatric experience. The standardized protocol included: (i) a preparation phase (5 min) of gentle stroking massage to the affected cervical region at a rate of approximately 60 strokes per minute to warm tissues and reduce infant distress; (ii) an SCM-targeted phase (10 min) incorporating pressing-kneading (approximately 120 repetitions per technique cycle), pushing, and grasping-lifting manipulation techniques applied along the SCM belly and attachments, with pressure graded to infant tolerance; and (iii) an acupoint stimulation phase (5 min) including Tianmen stimulation and related pediatric acupoint massage (approximately 100 circular strokes per acupoint). Techniques were standardized via a written protocol with photographic reference and a pre-study inter-rater reliability assessment among participating TCM practitioners (intraclass correlation coefficient > 0.80 for all techniques).

The functional exercise program was delivered by physical therapists and included: (i) visual tracking play with high-contrast toys to promote contralateral cervical rotation (3 sets of 10 repetitions per session, with target rotation angle progressed by 5° increments as tolerated); (ii) active-assisted cervical ROM exercises targeting lateral flexion and rotation toward the restricted side (3 sets of 8 repetitions per side, progressed to active-only as infant capacity improved); (iii) side-lying play to promote symmetrical head and trunk control (10-min sustained positioning periods, progressed from supported to unsupported); and (iv) prone positioning with graded support to promote head lifting and active cervical rotation (3 × 3-min intervals, progressed by reducing support surface height). Parents were trained during each session to perform a daily home program, including modified Tuina techniques (15 min), visual tracking play (10 minutes), positioning changes, prone play, and side-lying play. Caregivers documented home-program completion in daily logs and were followed up weekly by telephone or in person to check adherence and refine technique.

#### 3.5.2 Control group: conventional physical therapy

The control group infants received conventional physical therapy three times a week for 30 min per session for 12 weeks. Treatment sessions consisted of passive stretching of the SCM (cervical lateral flexion, rotation, and combined movements; 3 sets of 10 repetitions per direction per session), soft-tissue massage of the affected cervical region (10 min), and education about environmental positioning and caregiver instruction (5 min). Parents were taught a home program of passive stretching, positioning, and prone play, which was documented in a daily diary and reviewed at each session. It is important to acknowledge that the integrated group received substantially more therapist-contact time per session (45 min vs. 30 min), representing a 50% increase in direct treatment exposure. This constitutes a significant methodological limitation: the observed between-group differences in outcomes cannot be attributed specifically to the content of the interventions (Tuina therapy or functional exercise), because they may be partially or substantially explained by differences in the total dose of therapeutic contact, caregiver engagement, and coaching intensity. It is well established in rehabilitation research that therapist-contact time itself can influence outcomes independently of specific treatment content, through mechanisms such as motivation, reinforcement of home practice, and non-specific effects of therapeutic interaction. The magnitude of this confound cannot be estimated or statistically adjusted for in the current retrospective design, and all comparative findings must therefore be interpreted with this limitation explicitly in mind. Between-group differences should be considered as reflecting the combined package (integrated intervention including increased contact time) versus the standard care package, rather than as attributable to any specific therapeutic technique.

### 3.6 Outcome measures

Outcome assessors were not blinded to treatment allocation, as this was a retrospective review of routine clinical assessments. Standardized measurement procedures were applied during routine care, but the absence of blinded independent assessment is acknowledged as a potential source of measurement bias.

Primary outcomes were assessed at baseline and at 12 weeks: (1) Cervical ROM deficit was measured goniometrical with the infant supine, aligning the goniometer axis over the sternal notch, the stationary arm along the trunk midline, and the movable arm along the nasal direction during passive end-range cervical rotation; the ROM deficit was calculated relative to expected symmetrical rotation (2) SCM thickness ratio was measured by ultrasound as the ratio of affected-side to unaffected-side SCM thickness, with higher values indicating greater asymmetry (14). (3) Head tilt angle was measured by standardized photographic analysis as the angle between a vertical reference line and the craniofacial midline; multiple measurements were averaged when available.

Secondary outcomes included: clinical resolution (defined as simultaneous achievement of ROM deficit ≤ 5°, SCM thickness ratio ≤ 1.3, and head tilt angle ≤ 5°); time to clinical resolution (weeks); Muscle Function Scale (MFS) score at 12 weeks [ordinal scale 0–4, where 4 indicates age-appropriate lateral neck flexor function (15)]; parent-reported satisfaction (scored on a validated 50-point Likert-based scale, with higher scores indicating greater satisfaction); home-program adherence (percentage of prescribed sessions completed per caregiver logs); and adverse events.

### 3.7 Statistical analysis

Continuous variables are presented as mean ± standard deviation (SD) or median [interquartile range (IQR)] depending on distribution. Categorical variables are presented as frequencies and percentages. Between-group comparisons for continuous primary outcomes used independent-samples *t*-tests, with results reported as mean difference (95% CI) and Cohen’s *d* effect size. Ordinal and non-normally distributed continuous variables were compared using the Mann–Whitney U test. Categorical outcomes were compared using chi-square or Fisher exact tests, with results expressed as absolute risk difference (95% CI).

For continuous primary outcomes, change from baseline was calculated as:

Change score = 12-week value − baseline value

Between-group difference in change score and 95% CI was calculated using standard two-sample methods. Effect sizes were expressed as Cohen’s *d*, with |d| ≥ 0.2, 0.5, and 0.8 indicating small, medium, and large effects respectively. Given the exploratory, non-randomized design, p-values were interpreted descriptively and two-sided *p* < 0.05 was considered the threshold for exploratory significance. Because three primary outcomes were evaluated simultaneously, multiplicity is acknowledged and findings should not be interpreted as confirmatory. Subgroup analyses by severity, age group, and affected side were pre-specified as exploratory. Missing data were handled using available-case analysis with no imputation. All analyses were conducted in Python 3.11 (scipy 1.12, pandas 2.2). Additional supporting information is provided in the Supplementary material.

## 4 Results

### 4.1 Participant flow

**FIGURE 1:**
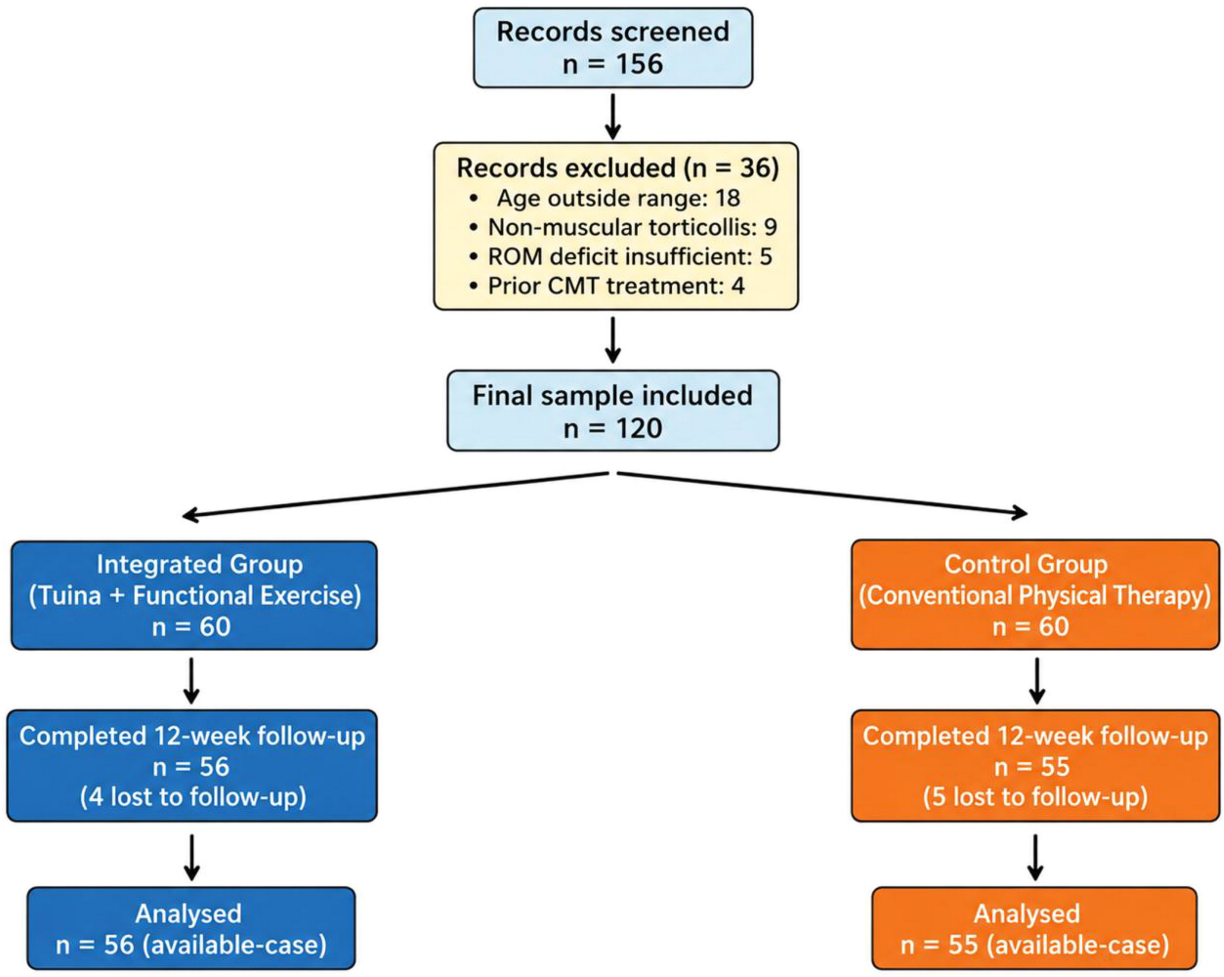
Participant flow diagram (STROBE—compliant). Records of 156 infants were screened; 36 were excluded. The final sample included 120 infants (60 per group). Complete 12-week data were available for 56 (integrated) and 55 (control) participants. Available-case analysis was applied.

From January to June 2024, the records of 156 infants with suspected or confirmed CMT were reviewed. Excluded were 18 infants because their age was outside the inclusion criteria, 9 because torticollis was due to non-muscular causes, 5 because their ROM deficit was less than 10 degrees, and 4 because they had been treated for CMT. A total of 120 infants (*n* = 60 per group) were retrospectively analyzed. Primary outcome data were available for 56 infants in the integrated group and 55 infants in the control group at 12 weeks. Infants withdrew due to family relocation, inability to schedule, or unspecified reason; none because of adverse events. An available-case analysis was conducted for the completers (*n* = 56 and *n* = 55). The participant screening, exclusion, allocation, and available-case analysis process is summarized in Figure 1.

### 4.2 Baseline characteristics

**FIGURE 2:**
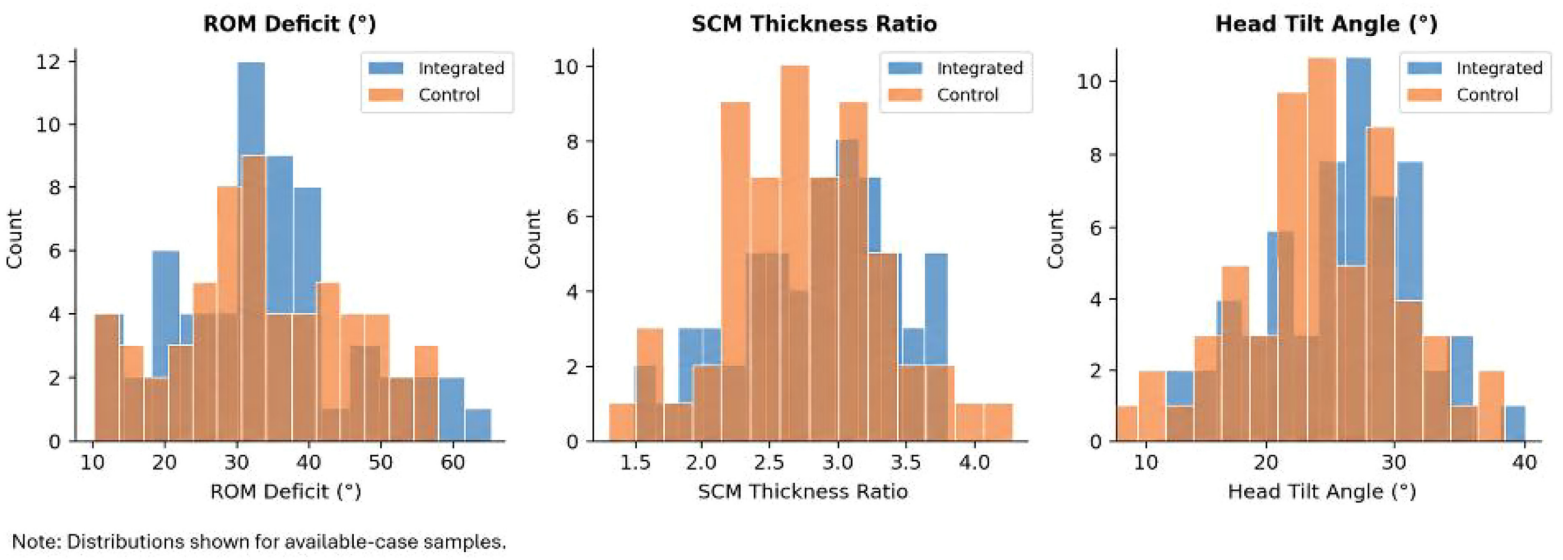
Baseline distribution of primary outcome variables by group. Histograms showing the baseline distribution of cervical ROM deficit, SCM thickness ratio, and head tilt angle in the integrated and control groups. Distributions were broadly comparable between groups.

Baseline characteristics were similar between groups across all measured variables (Table 1). No statistically significant between-group differences were observed for age (*p* = 0.90), sex (*p* = 0.69), affected side (*p* = 0.84), presence of SCM mass (*p* = 0.72), or baseline primary outcome values (ROM deficit *p* = 0.90; SCM ratio *p* = 0.32; head tilt *p* = 0.16). Similarity in measured baseline variables does not exclude unmeasured confounding given the non-randomized design. The baseline distributions of cervical ROM deficit, SCM thickness ratio, and head tilt angle were broadly comparable between groups, as shown in Figure 2.

**TABLE 1:** Baseline characteristics of study participants.

Characteristic	Integrated group (*n* = 60)	Control group (*n* = 60)	*p*-value
Age (months), mean ± SD	3.4 ± 1.5	3.6 ± 1.5	0.90
Male sex, n (%)	36 (60.0)	38 (63.3)	0.69
Right affected side, n (%)	33 (55.0)	34 (56.7)	0.84
SCM mass present, n (%)	31 (51.7)	29 (48.3)	0.72
Severe CMT at baseline, n (%)	28 (46.7)	29 (48.3)	0.88
ROM deficit, degrees (mean ± SD)	34.2 ± 12.1	33.8 ± 11.9	0.90
SCM thickness ratio (mean ± SD)	2.78 ± 0.56	2.71 ± 0.55	0.32
Head tilt angle, degrees (mean ± SD)	25.8 ± 6.0	24.9 ± 5.8	0.16
MFS score, median [IQR]	1 [0–2]	1 [0–2]	0.76

MFS, Muscle Function Scale (0–4; higher, better lateral neck flexor function). CMT, congenital muscular torticollis. Independent-samples *t*-test for continuous variables; chi-square for categorical. ROM, range of motion; SCM, sternocleidomastoid.

### 4.3 Primary outcomes at 12 weeks

**FIGURE 3:**
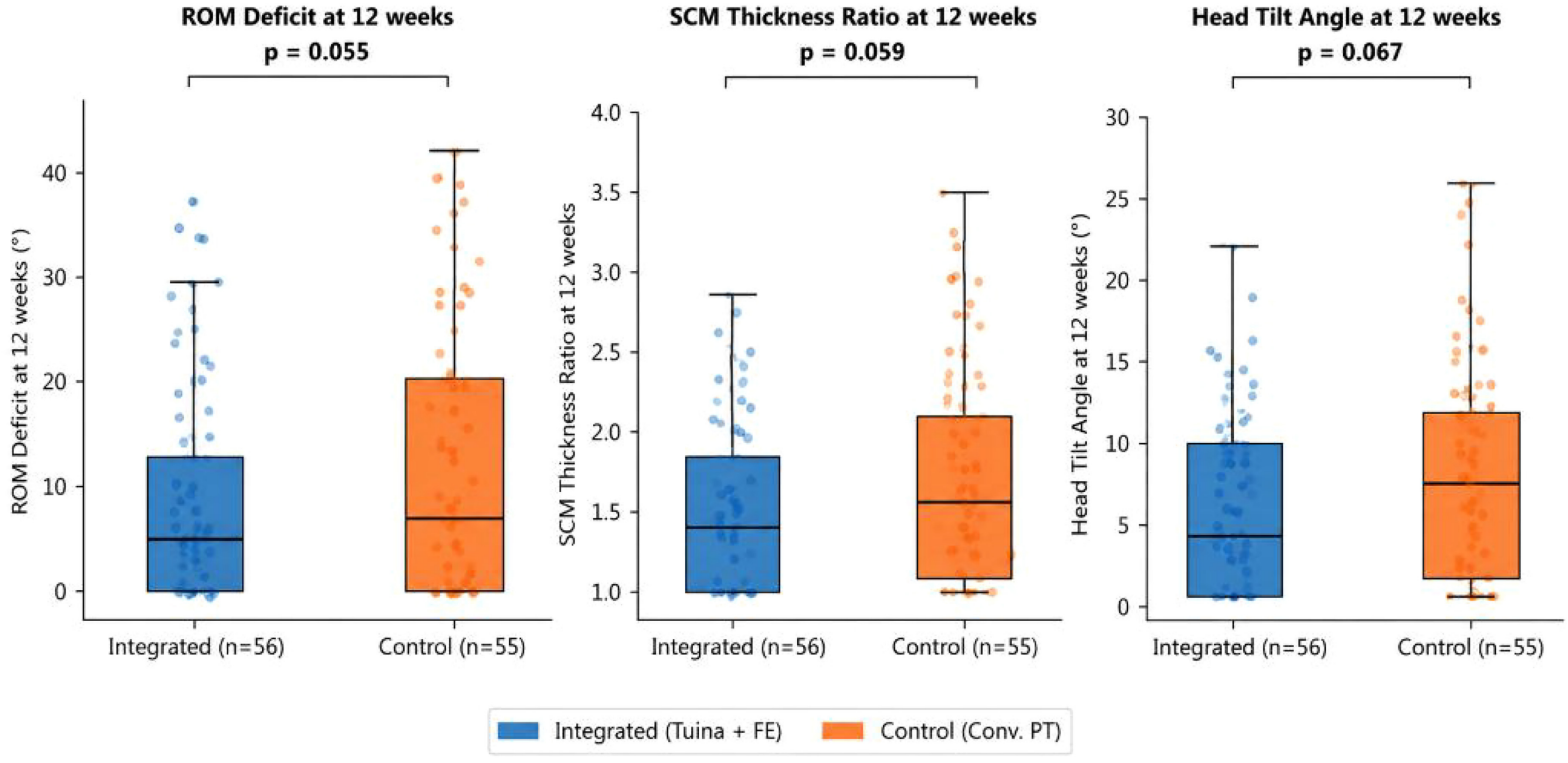
Primary outcome values at 12 weeks by group. Boxplots with individual data points for ROM deficit, SCM thickness ratio, and head tilt angle at 12 weeks. The integrated group (blue) demonstrated lower residual deficits than the control group (orange) across all primary outcomes. Individual points jittered for visibility.

**FIGURE 4:**
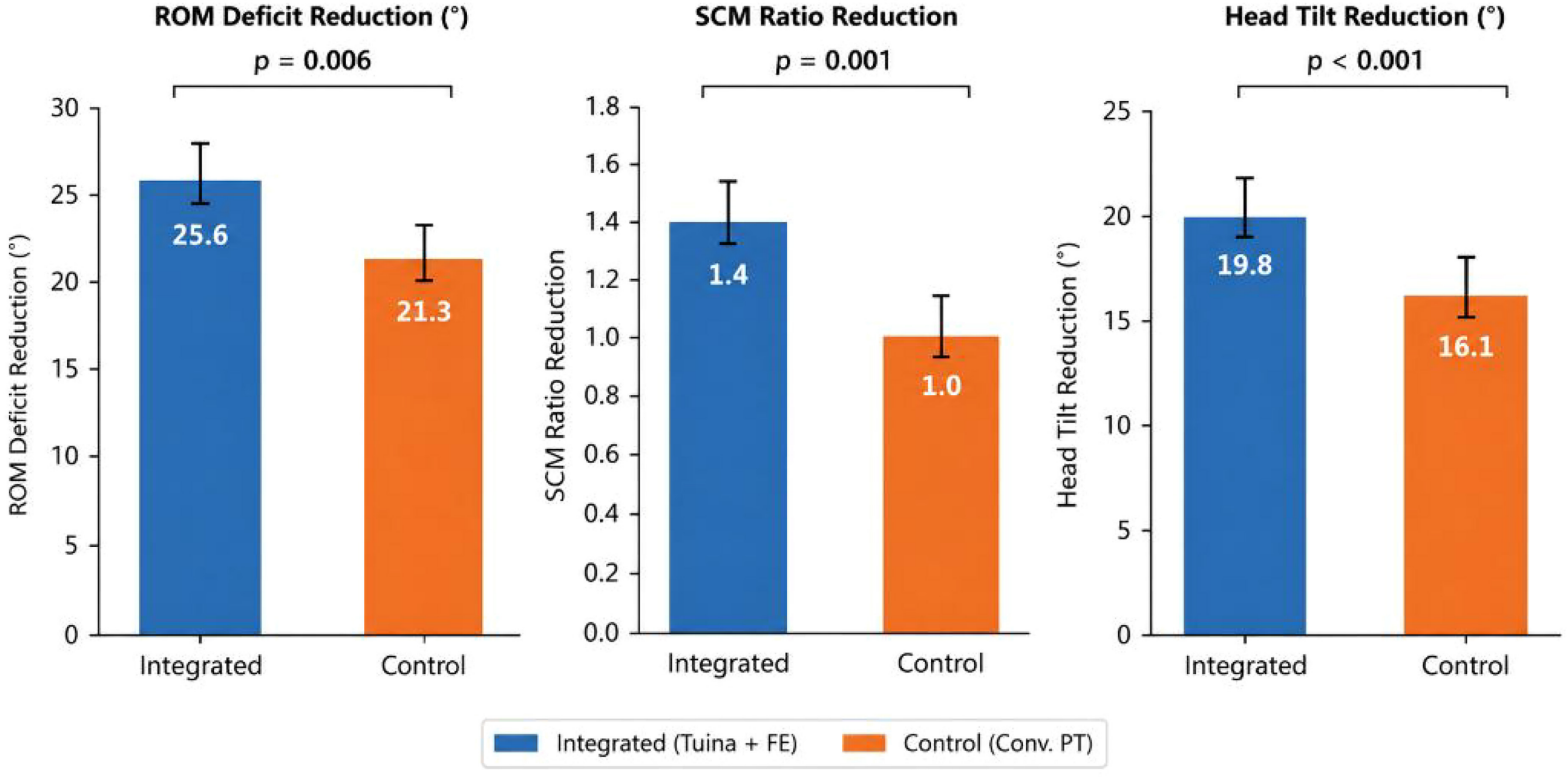
Mean change from baseline to 12 weeks. Bar charts (mean ± SEM) showing reductions from baseline in ROM deficit, SCM thickness ratio, and head tilt angle. Greater reductions were observed in the integrated group for all three outcomes. Significance brackets show exploratory *p*-values from independent-samples *t*-tests.

At 12 weeks, the integrated group demonstrated significantly greater mean change from baseline across all three primary outcomes compared with the control group. Full results are presented in Table 2 and illustrated in Figures 3, 4.

**TABLE 2:** Primary outcomes at 12 weeks.

Outcome	Integrated group (*n* = 56)	Control Group (*n* = 55)	Difference (95% CI)	*p*	*d*
12-week ROM deficit (°), mean ± SD	7.7 ± 9.9	11.9 ± 13.2	−4.1 (−8.3–0.04)	0.055	−0.35
Change in ROM deficit (°), mean ± SD	−25.9 ± 7.9	−21.4 ± 9.3	4.4 (1.3–7.5)	0.006	0.51
12-week SCM ratio, mean ± SD	1.50 ± 0.50	1.71 ± 0.66	−0.21 (−0.42–0.006)	0.059	−0.35
Change in SCM ratio, mean ± SD	−1.35 ± 0.49	−1.04 ± 0.55	0.31 (0.13–0.50)	0.001	0.60
12-week head tilt angle (°), mean ± SD	5.5 ± 5.6	7.6 ± 6.9	−2.1 (−4.4–0.13)	0.067	−0.34
Change in head tilt angle (°), mean ± SD	−19.8 ± 5.1	−16.1 ± 5.2	3.7 (1.9–5.6)	< 0.001	0.72

For endpoint values, between-group differences were calculated as integrated minus control. For change scores, differences were calculated using the absolute magnitude of improvement in the integrated group minus the absolute magnitude of improvement in the control group; therefore, positive values indicate greater improvement in the integrated group. 95% CI = 95% confidence interval; *d* = Cohen’s *d*. Available-case analysis: *n* = 56 (integrated), *n* = 55 (control). *p*-values from independent-samples *t*-tests; interpreted descriptively given exploratory design.

Cervical ROM deficit: The integrated group showed a mean reduction from baseline of 25.9 ± 7.9°compared with 21.4 ± 9.3° in the control group (between-group difference 4.4°, 95% CI 1.3–7.5, *t* = 2.81, *p* = 0.006, *d* = 0.51). The 12-week endpoint values were 7.7 ± 9.9° versus 11.9 ± 13.2° in the integrated and control groups respectively (between-group difference −4.1°, 95% CI −8.3–0.04, *p* = 0.055).

SCM thickness ratio: The integrated group demonstrated a mean reduction of 1.35 ± 0.49 compared with 1.04 ± 0.55 in the control group (difference 0.31, 95% CI 0.13 to 0.50, t = 3.29, *p* = 0.001, *d* = 0.60). The 12-week endpoint ratios were 1.50 ± 0.50 versus 1.71 ± 0.66 (difference −0.21, 95% CI −0.42–0.006, *p* = 0.059).

Head tilt angle: The integrated group showed a mean reduction of 19.8 ± 5.1°compared with 16.1 ± 5.2° in the control group (difference 3.7°, 95% CI 1.9–5.6, t = 3.95, *p* < 0.001, *d* = 0.72). The 12-week endpoint values were 5.5 ± 5.6° versus 7.6 ± 6.9° (difference −2.1°, 95% CI −4.4 to 0.13, *p* = 0.067).

### 4.4 Secondary outcomes

Clinical resolution (ROM deficit ≤ 5°, SCM ratio ≤ 1.3, and head tilt ≤ 5°) occurred in 7 of 56 infants (12.5%) in the integrated group and 4 of 55 (7.3%) in the control group (absolute risk difference 5.2%, 95% CI −5.0–15.4%, *p* = 0.527). The low overall resolution rates reflect the stringency of the composite resolution criterion and the 12-week follow-up duration.

**FIGURE 5:**
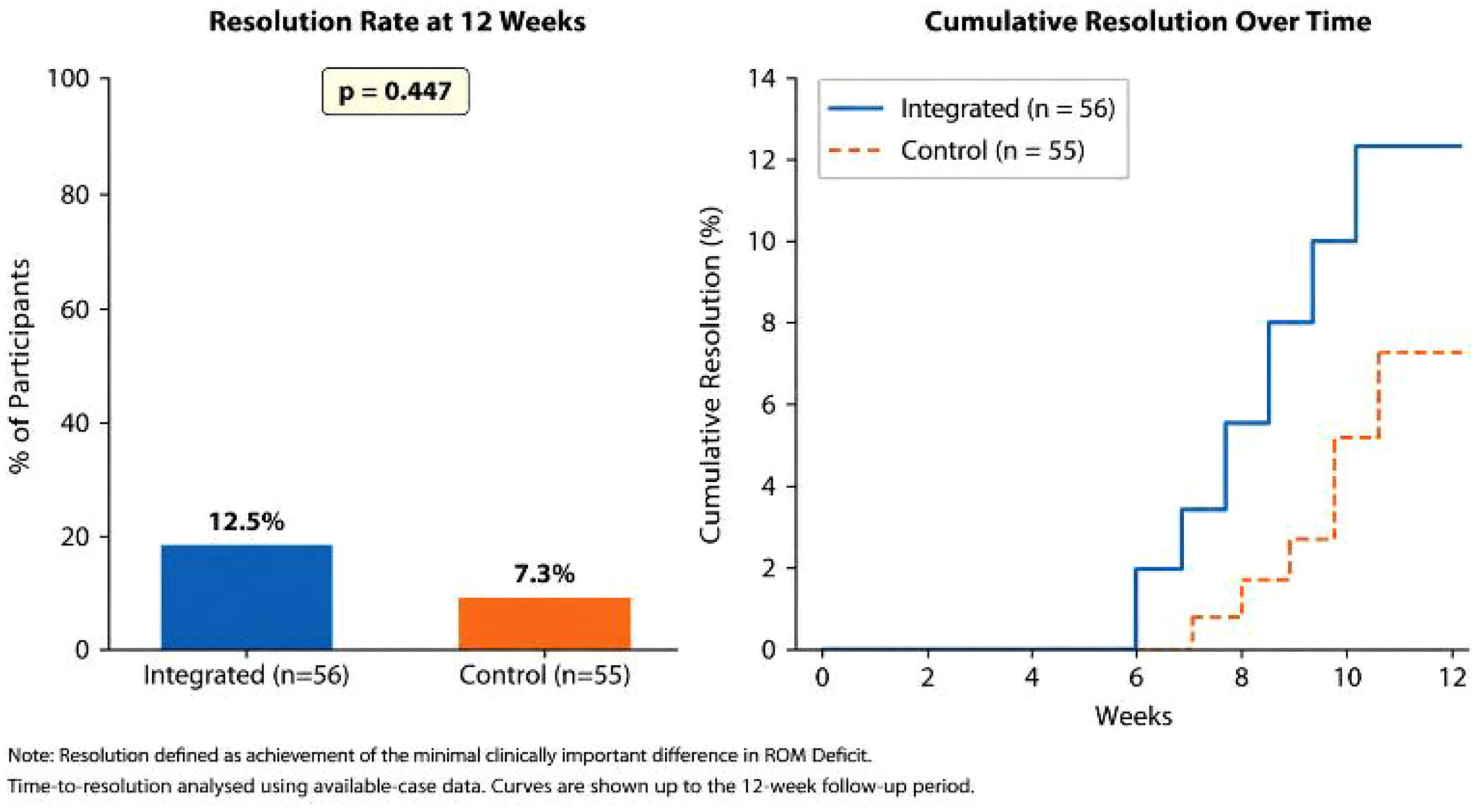
Clinical resolution rate and cumulative resolution curve. Left: Clinical resolution rates at 12 weeks (integrated 12.5% vs. control 7.3%; *p* = 0.527). Right: Median time to clinical resolution among resolved cases (integrated 9 weeks [IQR 8–10], *n = 7*; control 11 weeks [IQR 10–13], *n = 4*; *p* = 0.042). Time-to-resolution findings should be interpreted cautiously because they are based on a small number of resolved cases.

**FIGURE 6:**
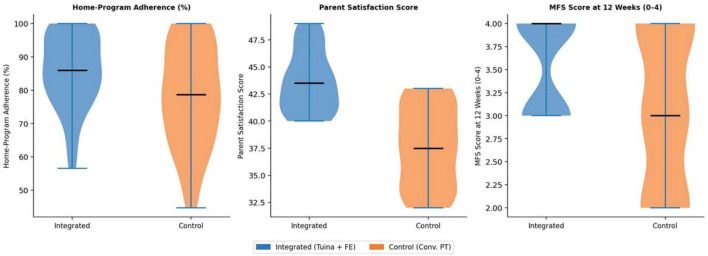
Secondary outcome distributions by group. Violin plots showing distributions of home-program adherence, parent satisfaction score, and MFS score at 12 weeks. The integrated group demonstrated higher values across all three secondary outcomes.

Median time to clinical resolution was shorter in the integrated group [9 weeks (IQR 8–10)] compared with the control group [11 weeks (IQR 10–13); Mann–Whitney U, *p* = 0.042]. However, these estimates were based on only 7 resolved cases in the integrated group and 4 in the control group and should therefore be interpreted cautiously. Home-program adherence was significantly higher in the integrated group (85.7 ± 11.2% vs. 78.0 ± 15.2%; difference 7.7%, 95% CI 3.0–12.5%, *p* = 0.002, *d* = 0.58). Parent-reported satisfaction was higher in the integrated group [median 44 (IQR 41–47) vs. 38 (IQR 35–41); *p* < 0.001]. MFS score at 12 weeks was also higher in the integrated group [median 4 (IQR 3–4) vs. 3 (IQR 2–4); *p* = 0.002]. Full secondary outcomes are presented in Table 3. Clinical resolution rates and cumulative time-to-resolution patterns are illustrated in Figure 5, while the distributions of home-program adherence, parent satisfaction, and MFS scores are shown in Figure 6.

**TABLE 3:** Secondary outcomes at 12 weeks.

Outcome	Integrated (*n* = 56)	Control (*n* = 55)	*p*-value
Clinical resolution, n/N (%)	7/56 (12.5%)	4/55 (7.3%)	0.527
Time to resolution, median (IQR) weeks	9 (8–10)	11 (10–13)	0.042†
Adherence,% (mean ± SD)	85.7 ± 11.2	78.0 ± 15.2	0.002*
Satisfaction score, median (IQR)	44 (41–47)	38 (35–41)	< 0.001†
MFS score at 12 weeks, median (IQR)	4 (3–4)	3 (2–4)	0.002†

MFS, Muscle Function Scale (0–4); satisfaction scale 0–50 (higher, more satisfied).

* Independent-samples *t*-test. † Mann–Whitney U test. Clinical resolution defined as simultaneous achievement of ROM deficit ≤ 5°, SCM ratio ≤ 1.3, and head tilt ≤ 5°.

### 4.5 Exploratory subgroup analyses

**FIGURE 7:**
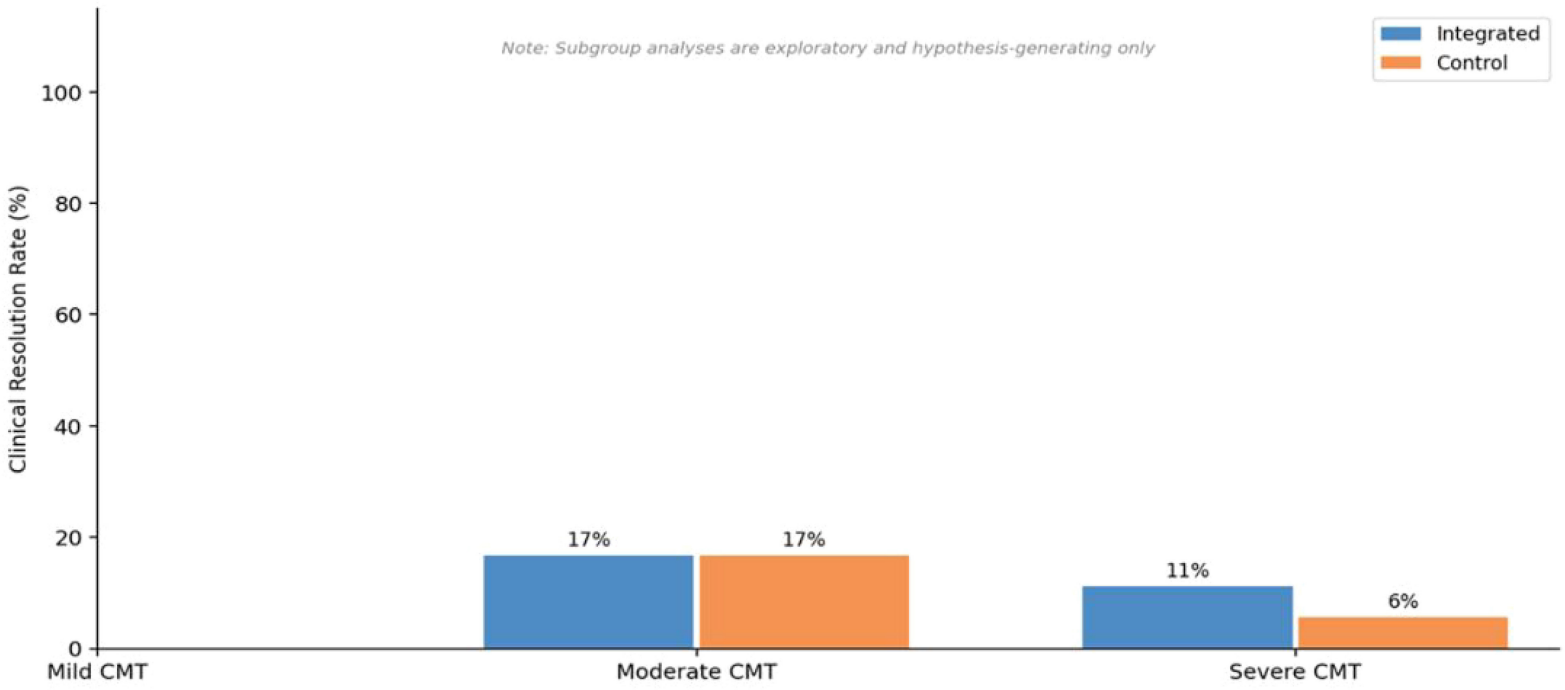
Exploratory subgroup analysis: resolution rate by baseline severity. Clinical resolution rates by baseline CMT severity (Mild, Moderate, Severe) for integrated and control groups. Subgroup analyses are exploratory only; no interaction testing was performed and multiple comparisons were not corrected.

Exploratory subgroup analyses suggested that treatment differences in resolution rate were broadly consistent across age categories, affected side, and presence or absence of an SCM mass. Possible variation by baseline severity was observed, with numerically larger differences among infants classified as severe at baseline (Figure 7). However, because the severity classification was study-specific, interaction analyses were exploratory and uncorrected for multiplicity, and these findings should not be interpreted as confirmatory evidence of effect modification.

### 4.6 Safety

**FIGURE 8:**
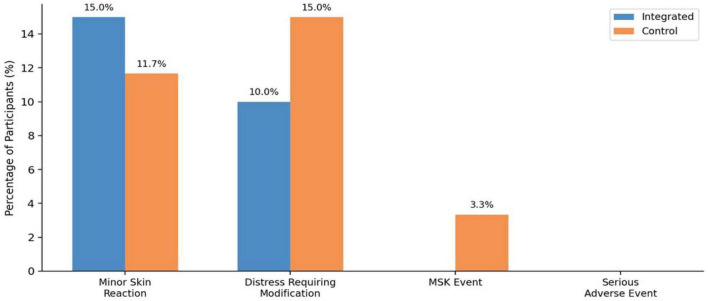
Adverse events by group. Percentage of participants with each adverse event by group. Minor skin reactions occurred in 13.3% of the integrated group and 6.7% of the control group; distress requiring session modification occurred in 8.3% and 18.3%, respectively; and one musculoskeletal event occurred in the control group. No serious adverse events occurred.

No serious adverse events were documented in either group during 12-week follow-up. Minor transient skin erythema occurred in 13.3% of the integrated group and 6.7% of the control group. Infant distress requiring session modification was documented in 8.3% of the integrated group and 18.3% of the control group. One musculoskeletal adverse event was recorded in the control group. No nerve injuries or withdrawals due to adverse events were documented (Table 4 and Figure 8).

**TABLE 4:** Safety outcomes.

Adverse event	Integrated (*n* = 60)	Control (*n* = 60)	*p*-value
Minor skin reaction, n (%)	8 (13.3)	4 (6.7)	0.22
Distress requiring session modification, n (%)	5 (8.3)	11 (18.3)	0.11
Musculoskeletal event, n (%)	0 (0.0)	1 (1.7)	1.00
Serious adverse event, n (%)	0 (0.0)	0 (0.0)	–
Withdrawal due to adverse event	0	0	–

*p*-values from chi-square or Fisher exact test as appropriate. – indicates no statistical test applicable.

## 5 Discussion

### 5.1 Principal findings

This retrospective exploratory study observed that infants with CMT who received Tuina therapy and functional exercise showed greater improvement from baseline in all three primary outcome measures (cervical ROM deficit, SCM thickness ratio, and head tilt angle) compared with infants receiving conventional physical therapy. Effect sizes for change scores ranged from small to medium (*d* = 0.51–0.72), with the greatest effect for head tilt angle. These observations are consistent with the hypothesis that a combined intervention targeting both structural and neuromotor aspects may offer additional benefit over conventional therapy; however, causal attribution is not warranted given the non-randomized retrospective design. Adherence, parent satisfaction, and MFS scores were also numerically higher for the integrated group, though these associations too may reflect non-specific effects of increased therapist contact and engagement rather than specific intervention components. A critical interpretive caution is that the integrated group received 50% more therapist-contact time per session (45 vs. 30 min). This unequal exposure represents a meaningful potential confounder: improvements observed in the integrated group may be partially attributable to the greater volume of therapeutic interaction, caregiver coaching, and home-program reinforcement, rather than to the specific content of Tuina or functional exercise. This confound cannot be disentangled in the current design, and it substantially constrains the inferential value of between-group comparisons.

However, there are some caveats. The retrospective, non-randomized design limits causal inference; differences may be due to selection bias, differing treatment volumes (45 vs. 30 min per session) or the motivation of parents, among other factors. Resolution rates were low (12.5% vs. 7.3%) due to the composite nature of the outcome measure and the 12-week follow-up period. This study should be viewed as exploratory.

### 5.2 Comparison with existing literature

The observed improvements in cervical ROM and SCM thickness ratio are broadly consistent with prior literature. Song et al. (14) demonstrated significant improvements in SCM thickness ratio and head rotation in a randomized trial of physical therapy. Chen et al. (8) and Kim et al. (9) reported that TCM massage and Tuina-based approaches improved ROM and resolution rates in systematic reviews, though with heterogeneous effect sizes. Tang et al. (10) reported similar directional findings in a retrospective comparative design. Lin et al. (11) further demonstrated improvement with massage combined with adjunctive electrotherapy. The current research builds on this literature by implementing a standardized combined protocol and longitudinal objective assessment in a natural clinical sample.

The observation of comparatively large treatment differences in relatively smaller infants with severe baseline CMT in the exploratory subgroup is consistent with some previous reports of poorer prognosis in severe cases but might indicate the potentially beneficial effect of multimodal intervention in cases with more severe structural impairment. This observation has to be formally confirmed by interacting with prospective participants.

### 5.3 Mechanistic interpretation

Mechanistic explanation of perceived benefits is hypothetical. The tuina treatment can help alleviate SCM stiffness and enhance local microcirculation, which might help to increase tissue extensibility and remodeling (8, 10). Neuromotor re-education and caregiver-mediated practice may facilitate active motor control, head-righting, and postural symmetry with the use of functional exercise (1, 15). Combination of these methods can result in bidirectional facilitation, that is, better tissue extensibility to increase active training engagement, and repeated motor training to consolidate structural benefits, but these interpretations are speculative without mechanistic data.

### 5.4 Strengths and limitations

The strengths of this study are a clear clinical sample, even group sizes, several objective outcome measures that were measured longitudinally, and adherence, satisfaction, and functional outcomes data which offer a comprehensive real world assessment. Ecological validity is enhanced with the use of real clinical data.

Several important limitations are acknowledged. First, the retrospective non-randomized design introduces a substantial risk of selection bias and confounding, and precludes causal inference; observed differences are associative only. Second, outcome assessors were not blinded to treatment allocation, which may have introduced systematic measurement bias, particularly for outcomes with a subjective component. Third, and critically, the integrated group received 50% more therapist-contact time per session (45 vs. 30 min). This imbalance means that observed between-group differences cannot be attributed specifically to Tuina therapy or functional exercise, because increased therapeutic exposure, caregiver engagement, and reinforcement of home practice may independently drive improved outcomes; this confound cannot be statistically controlled in the current design and should be addressed explicitly in any future prospective study through contact-time equalization or an active control group with equivalent session duration. Fourth, the absence of Tuina-only and functional exercise-only comparison arms prevents estimation of the independent contribution of each intervention component. Fifth, the single-center retrospective design limits external generalizability. Sixth, the 12-week follow-up duration is insufficient to evaluate long-term outcomes or resolution trajectories. Seventh, the composite resolution criterion was stringent, resulting in low resolution rates in both groups. Eighth, the study-specific severity classification was not externally validated and may influence subgroup interpretation. Ninth, unmeasured confounders such as caregiver health literacy, socioeconomic status, and motivation may have differed systematically between groups given non-randomized allocation. In addition, time-to-resolution estimates were based on a very small number of resolved cases and should therefore be interpreted cautiously.

### 5.5 Future directions

Future studies must focus on prospective multicentre randomized controlled trials that use blinded outcome assessment, equal therapist-contact time of all arms, standardized intervention protocols, pre-specified primary outcomes, and longer follow-up. Factorial designs between Tuina-only, functional exercise only, combined and conventional arm of therapy would enable estimation of component-specific effects. Mechanistic research that integrates ultrasound elastography, biomechanical and neurophysiological outcomes are required to explain mechanisms behind treatment effects. Implementation research is needed to determine the feasibility, training needs, and implementation obstacles in various clinical and resource environments.

## 6 Conclusion

This retrospective exploratory study demonstrated that infants with congenital muscular torticollis who received combined Tuina therapy and functional exercise showed significantly greater improvements in cervical range of motion, SCM thickness ratio, and head tilt angle over 12 weeks compared with those receiving conventional physical therapy, with small-to-medium effect sizes. Secondary outcomes including home-program adherence, parent satisfaction, and functional performance also favored the integrated approach. However, given the retrospective non-randomized design, unequal therapist-contact time, and absence of blinded outcome assessment, these findings should be considered associative and hypothesis-generating. High-quality prospective randomized controlled trials with standardized treatment intensity and blinded assessment are required before definitive clinical recommendations can be made.

## Data Availability

The datasets presented in this article are not readily available because the study was based on retrospective anonymized clinical records subject to patient privacy and institutional data-governance restrictions. De-identified aggregate data may be made available by the corresponding author upon reasonable request and after institutional approval. Requests to access the datasets should be directed to the corresponding author.
